# The Treatment Strategy for the Atrial Septal Defect in the Presence of Severe Pulmonary Hypertension

**DOI:** 10.3390/medicina58070892

**Published:** 2022-07-02

**Authors:** Evelina Zarambaitė, Grytė Ramantauskaitė, Aušra Krivickienė, Adakrius Siudikas, Skaidrius Miliauskas, Eglė Ereminienė

**Affiliations:** 1 Medical Academy, Lithuanian University of Health Sciences, 44307 Kaunas, Lithuania; evelina.zarambaite@gmail.com; 2 Department of Cardiology, Medical Academy, Lithuanian University of Health Sciences, 44307 Kaunas, Lithuania; krivickiene.ausra@gmail.com (A.K.); egle.ereminiene@kaunoklinikos.lt (E.E.); 3 Cardiology Institute, Lithuanian University of Health Sciences, 44307 Kaunas, Lithuania; 4 Department of Cardiac Surgery, Medical Academy, Lithuanian University of Health Sciences, 44307 Kaunas, Lithuania; adakrius.siudikas@kaunoklinikos.lt; 5 Department of Pulmonology, Medical Academy, Lithuanian University of Health Sciences, 44307 Kaunas, Lithuania; skaidrius.miliauskas@kaunoklinikos.lt; 6 Kaunas Society of Cardiology, 44307 Kaunas, Lithuania

**Keywords:** congenital heart disease, pulmonary arterial hypertension, atrial septal defect, “treat and repair” strategy

## Abstract

Atrial septal defect is one of the most common congenital heart diseases in adults. The defect often leads to volume overload in the right heart coupled with the potential risk of right heart failure and pulmonary arterial hypertension. These conditions lead to worsening in quality of life, decrease in physical capacity, and even to fatal outcomes. The main strategy for treatment of atrial septal defect is a transcatheter or surgical closure of the defect, but in patients with severe pulmonary arterial hypertension, it is recommended to manage pulmonary arterial hypertension and after that treat the defect invasively. This strategy is called “treat and repair” strategy. We present an illustrative case report of management and treatment of atrial septal defect, complicated with severe pulmonary arterial hypertension. In this case, surgical closure of the defect was contraindicated because of the high pulmonary vascular resistance. Therefore, the “treat and repair” strategy was approached. After specific medical treatment of pulmonary arterial hypertension, surgical closure of the defect was chosen and proven successful.

## 1. Introduction

Atrial septal defect (ASD) is a congenital heart defect, constituting 10–15% of all congenital heart diseases [1]. Prognosis and clinical manifestation depend on the size and localization of the defect. As a result of the improved health services, most of the newborns with congenital ASD successfully reached adult age [2].

Pulmonary arterial hypertension (PAH) affects approximately 10% of patients with congenital heart disease, impacting their quality of life and survival [3]. In the case of ASD, the abnormal blood shunt from left to right occurs and the overload of the right heart and pulmonary artery (PA) emerges. The pressure in PA increases and in most of the cases PAH develops, but also hyperkinetic pulmonary hypertension can occur, which is reversible after the closure of the defect and has a good long term survival [4]. Rarely, in about 1% of patients with congenital ASD, Eisenmenger’s syndrome occurs because of the progression of PAH [5]. This syndrome emerges as there is a highly increased resistance of pulmonary arteries when the pressure in the right atrium (RA) becomes higher than in the left atrium (LA). Prevention of this syndrome is an important target of septal defects management, as PAH is associated with eightfold increased probability of functional limitations [5].

The main therapy of ASD: transcatheter defect closure or surgical repair of the defect. In the presence of PAH, it is recommended that pulmonary vascular resistance (PVR) be less than five Wood units (WU) to approach interventional or surgical treatment [2]. If PVR is higher than 5 WU, it is recommended to approach the ‘treat and repair’ strategy—treating PAH with medications first and after reaching a PVR with less than 5 WU, to repair the defect invasively [2,6]. Despite that, the prevalence of PAH remains high even after the closure of the defect [7].

In this clinical case, we present a 56 years old patient, with congenital ASD, complicated with severe PAH, diagnostics and treatment approach.

## 2. Clinical Case

A 56-year-old male was hospitalized at the Hospital of Lithuanian University of Health Sciences, Kaunas clinics with a six-month history of progressive dyspnea that occurred during minor physical activity. The patient had no comorbidities or heart diseases in his previous medical history. Cardiac auscultation revealed rhythmic heartbeats with a mid-systolic ejection murmur in Erb‘s point.

At the time of initial diagnosis, the patient had III functional class heart insufficiency classified by the New York Heart Association (NYHA). The systolic blood pressure (BP) of the patient was 136 mmHg, diastolic BP 82 mmHg, heart rate was 66 beats/min. A 6 min distance test estimated the moderate physical capacity (367 m). Blood oxygen saturation (SpO2) before physical activity was 88% and after activity saturation dropped to 78%. 

The results of laboratory tests were as follows: the concentration of natriuretic peptides (NTproBNP) was increased up to 2228 ng/L (normal ranges (n) less than 125 ng/L), secondary erythrocytosis (erythrocytes 5.52 × 10^9^/L), increased concentration of hemoglobin (183 g/L), slightly higher liver enzymes (alanine aminotransferase (ALT) 61 IU/L, aspartate transaminase (AST) 54 IU/L), slightly reduced renal function (concentration of creatinine—88 μmol/L, estimated glomerular filtration rate (eGFR)—85 mL/min).

**Figure 1:**
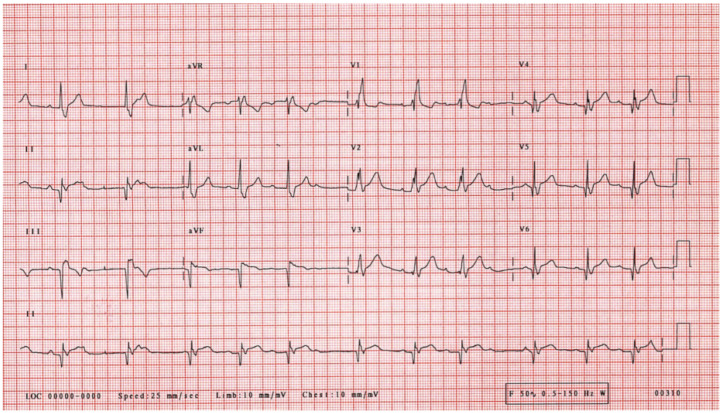
The electrocardiogram shows sinus rhythm, right bundle branch block, and right heart overload.

**Figure 2:**
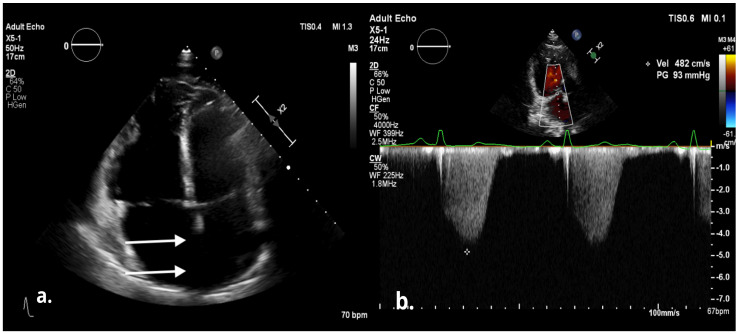
Transthoracic echocardiography imaging shows the dilatation of right heart chambers, atrial septal defect (marked by the arrows) (**a**) and tricuspid regurgitation with high maximal regurgitant velocity (**b**).

**Figure 3:**
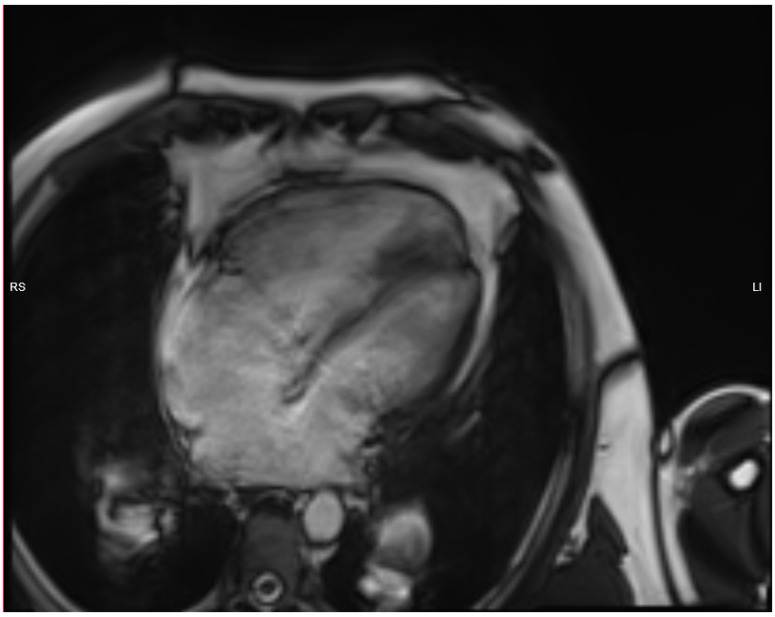
Magnetic resonance imaging shows the overload of the right heart and large secondary ASD (4.2 × 5.4 cm).

**Figure 4:**
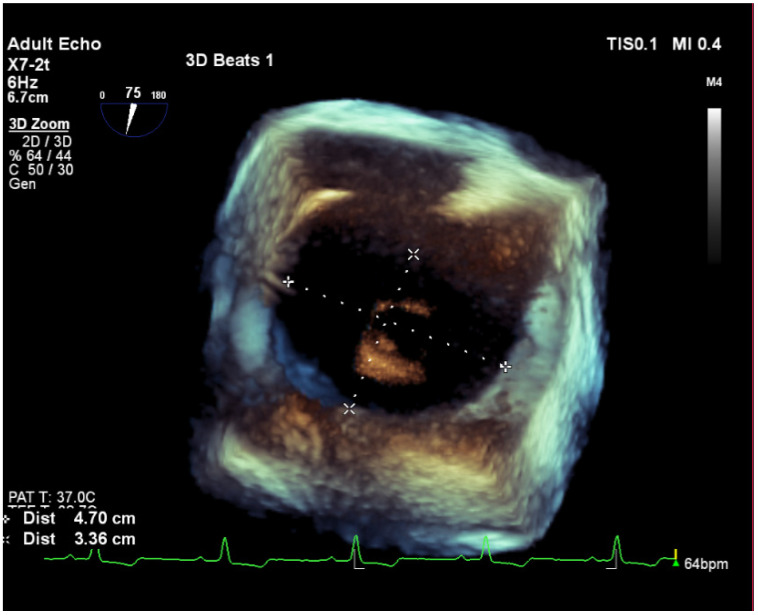
3D Transesophageal echocardiography showing the size of the atrial septal defect.

The electrocardiogram showed sinus rhythm, signs of right heart overload, and right bundle branch block (Figure 1). Moreover, transthoracic echocardiography revealed the hemodynamically significant ASD with right heart chamber dilatation, PAH (systolic PA pressure—100 mmHg, the mean PA pressure—50 mmHg), and preserved systolic left ventricular function (ejection fraction was 50%), although there was impaired relaxation in diastolic left ventricular function. Furthermore, the severe eccentric tricuspid valve (TV) regurgitation (maximal gradient 92.16 m/s) because of TV anterior cusp prolapse was identified (Figure 2). Central venous pressure was 6 mmHg. To better assess the size and localization of ASD, the overload of the right heart, Qp/Qs ratio and possible other congenital heart defects, the transesophageal echocardiography, and heart magnetic resonance (MR) was performed (Figure 3 and Figure 4). The findings were as follows: a large secundum ASD (4.2 × 5.4 cm), dilated pulmonary trunk (47 mm), and pulmonary branches (36 mm). The overload of the right heart (right ventricular (RV) end-diastolic volume index—109 mL/m^2^, right atrial volume—57 mL/m^2^), decreased RV systolic function (ejection fraction—48%) and no other congenital heart defects were found. The lung perfusion scintigram showed no evidence of pulmonary embolism.

The invasive hemodynamics study was performed and revealed a hemodynamically significant ASD, causing a severe pulmonary volume overload and significant precapillary pulmonary hypertension (systolic PA pressure was 86 mmHg, mean PA pressure 50 mmHg, mean pulmonary capillary Wedge pressure 7 mmHg) with high PVR (11–12 WU). 

The multidisciplinary team council decided that surgical repair of the defect was contraindicated because of the high PVR at the moment. Specific PAH treatment with phosphodiesterase-5 inhibitor sildenafil was prescribed to reduce PVR and an invasive hemodynamics study six months after specific PAH treatment to was recommended to reassess surgical treatment options. 

After six months of specific PAH monotherapy with sildenafil (dosing 20 mg 3 times a day), an invasive hemodynamics study was repeated—significant precapillary PAH (mean systolic PA pressure 50 mmHg) remained but the PVR decreased from 11 to 8 WU. Despite the persistence of relatively high PVR, the multidisciplinary team council decided that surgical treatment was amenable for this patient and transcatheter closure of the defect was contraindicated because of the large (over 4 cm) defect size.

The surgical TV repair, annuloplasty and ASD suturing with an autopericardial patch (oriented from the left atrium) were performed. The postoperative period was complicated by respiratory failure due to pulmonary hypertension and pneumonia. Antibiotics for the treatment of pneumonia were prescribed. Specific treatment of PAH was adjusted: additional endothelin receptor antagonist ambrisentan and prostacyclin analogue inhaled iloprost were prescribed together with phosphodiesterasis-5 inhibitor—sildenafil. Diuretics and mineralcorticoid receptor antagonists were prescribed for the treatment of heart failure.

In 12 months after cardiac surgery (ASD closure and TV repair) the patient’s condition improved after the successful treatment—shortness of breath and natriuretic peptide levels (NT-proBNP dropped from 2228 to 350 ng/L) reduced. Moreover, the results of the 6-min distance test improved (from 367 to 477 m.) [Table 1]. Furthermore, there was an improvement in NYHA functional class—it reduced from III functional class to II. Echocardiography revealed positive hemodynamical changes: decreased right ventricular dilation (RV end-diastolic diameter—from 53 to 43 mm), PAH (mean systolic PA pressure—from 50 to 40 mmHg), and improved global RV systolic function (FAC increased from 20% to 29%) [Table 2].

The further recommendation was to continue the specific PAH treatment with sildenafil and ambrisentan. The patient was regularly consulted by a cardiologist every three months, to reassess further changes in PAH.

## 3. Discussion

In patients diagnosed with severe PAH (systolic PA pressure >70% of systemic pressure, PVR > 8 WU and no response to vasodilators), ASD closure is not indicated. It is difficult to predict the outcomes due to advanced pulmonary vascular remodeling. To apply a treatment strategy to this group of patients, it is necessary to evaluate the resistance of the pulmonary vessels. According to the latest congenital heart defects treatment guidelines, in patients with PVR < 5 WU, closing ASD is a safe procedure followed by systolic PA pressure reduction and regression of the symptoms [2]. In patients with PVR ≥ 5 WU, optimal specific treatment of PAH is primarily required, re-evaluation of hemodynamics is mandatory and surgical/transcatheter ASD closure is considered only if PVR falls below 5 WU in presence of significant left to right shunt. The ‘treat and repair’ strategy can reduce perioperative mortality and improve outcomes in patients with high PVR [8]. In this clinical case, the patient was diagnosed with an increased PVR (11 WU) and was given optimal medical therapy with a phosphodiesterase-5 inhibitor sildenafil. Six months later, the invasive hemodynamics study was repeated and revealed positive but not desired hemodynamical changes—PVR decreased to 8 WU. In such cases, insufficient reduction of PVR is required to continue specific medical treatment of PAH, but an aggressive treatment strategy—surgical closure of ASD—was chosen. The strategy was proven successful and a positive dynamic was observed after 12 months.

‘Treat and repair’ strategy may extend the window of operability in carefully selected patients with borderline hemodynamics. Arvind et al. have demonstrated a few cases with ASD and markedly elevated PVR that has shown unequivocal benefits after specific treatment of PAH and successful clinical outcomes after closure [6]. There are additional benefits of the closure of ASD even if the PVR is high: after surgical closure of ASD, the process of reverse remodeling starts. Several authors reported significant reductions in RA and RV diameters six months after surgical ASD closure [9,10,11]. An improvement of tricuspid annular plane systolic excursion (TAPSE) parameters in transthoracic echocardiography after the closure of the defect is also seen—it indicates an improvement of right ventricular function [11]. Nevertheless, in long-term follow-up improvement in regurgitation of the tricuspid valve is also seen, because of the reverse remodeling that occurs after the closure, and also, because of tricuspid valve repair itself [12].

Although the ‘treat and repair’ strategy may reduce the perioperative mortality of patients with shunt and raised PVR, the long-term outcome needs to be carefully investigated [6]. Some authors have shown that PAH may persist or even increase [13] and persistent elevated PA pressure can occur after ASD closure [12]. PAH after repair of the congenital heart disease is associated with increased morbidity and mortality, and also worsening of patient’s quality of life [7]. Persistence or subsequent development of PAH after defect closure surgery is affected by several factors. Gabriels et al., revealed age to be an independent predictor of persisted PAH after defect closure surgery [14]. Older patients had a worse clinical outcome after defect closure due to previous chronic overflow and volume overload on pulmonary circulation [14]. Moreover, patients with congenital heart disease who develop PAH after shunt closure have a worse prognosis than patients not undergoing shunt closure [15]. 

Whereas successful shunt repair is often considered a cure, many patients lose follow-up after surgical repair, but these patients require regular monitoring and re-evaluation due to the possibility of developing or aggravation of PAH. Even when the congenital defect is repaired at an early age, the late risk of PH development, in absence of left-sided heart disease, is >15% and has a significant impact on survival [7].

Close monitoring and follow up visits are necessary not only because of the increased risk of PAH development but also because of the possible long-term complications and states related to ASD and its closure. Complications might be sinus node dysfunction, progression of heart failure and mitral valve regurgitation [16]. Furthermore, the risk of stroke remains increased for these patients even after the closure of the defect [17]. Proper lifestyle and optimal medical therapy are strongly recommended for these patients. Furthermore, almost all patients need to continue specific medical therapy for PAH after shunt closure [6]. 

If patients have severe PAH, the operative risk is high, and the closure of the defect would result in deterioration of hemodynamics and worsening of symptoms—the closure of ASD is not recommended [18]. In the case of our patient, even when high PVR persisted (although a decrease from 11 to 8 WU was observed) after adequate specific medical treatment, a decision was made to operate on this patient expecting good results from the surgery. The positive dynamics were confirmed as further follow-up visits showed a reduction in symptoms and an improvement in quality of life.

## 4. Conclusions

In conclusion, the initial administration of specific PAH therapy may extend the treatment for carefully selected patients with significant ASD-induced precapillary PAH and improve the treatment options for interventional/surgical ASD. Each decision on the patient’s suitability for interventional, surgical or medical treatment must be individual and a multidisciplinary team of specialists (cardiologist, cardiac surgeon, interventional cardiologist, pulmonologist) must be involved in the decision-making process, and the regular follow-up monitoring is very important after the closure of the defect. 

## Figures and Tables

**Table 1:** Clinical and laboratory results before and after surgical ASD treatment.

	December 2019		July 2020		August 2021	
**NT-proBNP conc. (ng/L); N < 125**	2228		299		350	
**6-min distance test (m)**	367		380		477	
**SpO_2_ before and after 6-min distance test (%)**	88	78	-	-	98	94

NT-proBNP—N-terminal-proB-type Natriuretic Peptide.

**Table 2:** Echocardiography results before and after surgical treatment and follow-up.

Date	July 2020	July 2020 (before Surgical Treatment)	July 2020 (after Surgical Treatment)	August 2021
**LVEDD (mm)**	36	38	44	44
**RVEDD (mm)**	58	53	48	42
**RV S’ (cm/s)**	7.8	9.7	8.8	10.7
**RV FAC (%)**	17	23	26	36
**Max PA pressure (mmHg)**	100	95	70	55
**PAAT (ms)**	60	70	80	85

LVEDD—left ventricle end-diastolic volume. RVEED—right ventricle end-diastolic volume. RV S’—right ventricle systolic velocity. RV FAC—right ventricle fractional area change. PA—pulmonary artery. PAAT—pulmonary artery acceleration time.

## Data Availability

Not applicable.
